# Efficient Semi-Quantum Secure Multi-Party Summation Protocol Based on Cancelable Random Masks and Its Applications

**DOI:** 10.3390/e28070716

**Published:** 2026-06-23

**Authors:** Dan Wang, Diedie Yang, Haibin Wang

**Affiliations:** 1 School of Internet of Things and Artificial Intelligence, Wuxi Vocational College of Science and Technology, Wuxi 214028, China; wangdan@wxsc.edu.cn; 2 School of Computer Science, School of Software, Nanjing University of Information Science and Technology, Nanjing 210044, China; 3 School of Electronic and Information Engineering, Anhui Jianzhu University, Hefei 230601, China; diedieyang@stu.ahjzu.edu.cn

**Keywords:** quantum secure multi-party summation, semi-quantum protocol, quantum Fourier transform, cancelable mask

## Abstract

Quantum Secure Multi-party Summation (QSMS) is a fundamental primitive of Quantum Secure Multi-party Computation (QSMC), enabling multiple participants to jointly compute the sum of their private inputs without disclosing individual data. However, most existing QSMS protocols require all participants to possess full quantum capabilities and often rely on pre-shared keys, auxiliary mask transmission, or multiple trusted third parties, resulting in high communication overhead and limited practicality. To address these limitations, we propose an efficient Semi-Quantum Secure Multi-party Summation (SQSMS) protocol based on *d*-dimensional *n*-particle entangled states. By exploiting the global correlation properties of high-dimensional entangled states, the proposed protocol generates correlated random masks directly from quantum measurement outcomes. These masks cancel automatically during the aggregation process, eliminating the need for additional mask distribution and transmission. Compared with existing QSMS schemes, the proposed protocol reduces communication overhead, improves quantum efficiency, and avoids reliance on pre-shared keys or multiple trusted third parties. Moreover, only simple measurement operations are required from classical participants, making the protocol more practical for semi-quantum environments. We further provide formal correctness and security analyses of the proposed protocol and conduct quantum circuit simulations using the IBM Qiskit platform to demonstrate its feasibility. Moreover, based on the proposed summation protocol, we design several extended application protocols, including anonymous voting, anonymous auction, and anonymous ranking, which further illustrate the scalability and practical applicability of the proposed scheme.

## 1. Introduction

With the rapid development of cloud computing, big data, and artificial intelligence, data have become a critical strategic resource for economic activities, social services, and technological innovation. However, the increasing value of data is accompanied by growing risks of privacy leakage and security threats in cross-institutional data collaboration. In practical scenarios such as financial risk control, collaborative medical diagnosis, joint statistical analysis, and government information sharing, multiple entities are often required to jointly perform computational tasks without exposing their private data. This demand for “data usability without data disclosure” has driven the development of Secure Multi-party Computation (SMC). SMC enables multiple participants to jointly compute a target function without relying on a fully trusted third party, while ensuring that each participant’s private input remains confidential. Since Yao [1] introduced the classical “millionaire problem” and proposed the two-party secure computation framework, Goldreich et al. [2] further proved that any polynomial-time computable function can be securely realized in multi-party settings, thereby laying the theoretical foundation of SMC. After decades of development, SMC has evolved into several representative technical paradigms, including garbled circuits [3], secret sharing [4,5], oblivious transfer [6,7], and homomorphic encryption [8,9,10]. These techniques have demonstrated significant application value in privacy-preserving machine learning [11], anonymous voting [12,13], and secure statistical analysis [14].

Nevertheless, most classical SMC schemes rely on computational hardness assumptions, such as integer factorization and discrete logarithm problems. Their security fundamentally depends on the computational limitations of classical computers. With the rapid advancement of quantum computing, especially the emergence of Shor’s algorithm [15], which can solve integer factorization and discrete logarithm problems in polynomial time on ideal quantum computers, traditional public-key cryptosystems such as RSA face severe security threats. Consequently, constructing Secure Multi-party Computation mechanisms suitable for quantum environments has become an important research direction, leading to the development of QSMC. Benefiting from fundamental quantum mechanical properties, including the no-cloning theorem, quantum entanglement, and measurement disturbance, quantum cryptography provides a new framework for achieving information-theoretic security beyond classical computational assumptions. The BB84 quantum key distribution protocol proposed [16] demonstrated the intrinsic security advantages of quantum communication and established the theoretical foundation for subsequent quantum cryptographic protocols.

QSMS is an important branch of QSMC, aiming to securely compute the summation of private inputs without revealing participants’ confidential information. By exploiting quantum physical properties, QSMS can theoretically achieve information-theoretic security and has therefore attracted considerable attention in privacy-preserving computation research. However, existing QSMS protocols still face significant practical challenges. Most current schemes [17,18,19,20,21,22,23,24,25] assume that all participants possess full quantum capabilities, including quantum state preparation, unitary operations, and joint measurements. Given the high cost and limited accessibility of quantum hardware, such assumptions are difficult to satisfy in practical implementations. Furthermore, some protocols rely heavily on high-dimensional quantum systems or multi-particle entangled states [17,22,25] whose preparation, transmission, and error correction require substantial physical resources, resulting in increased communication overhead and system complexity. These limitations motivate the exploration of more practical quantum computation models that balance security and implementation feasibility.

To reduce the quantum capability requirements for ordinary users, Boyer et al. [26,27] proposed the concept of semi-quantum computation, where only a subset of participants possesses full quantum capabilities, while the remaining participants are restricted to simple operations such as reflecting qubits or measuring and resending qubits under fixed bases. Based on this idea, Semi-Quantum Secure Multi-party Computation (SQSMC) has gradually emerged as an important research direction in quantum privacy-preserving computation. In the field of SQSMS, Zhang et al. [28] first introduced the semi-quantum paradigm into multi-party summation in 2021 and proposed a three-party semi-quantum summation protocol. Using single photons as information carriers, the protocol employs GHZ-basis measurements to verify the behavior of the trusted third party and achieve secure summation of private inputs. Subsequently, Ye et al. [29] proposed a two-party semi-quantum summation protocol based on logical qubits, which improves resistance against collective decoherence noise and enhances privacy protection. In 2024, Tian et al. [30] designed three semi-quantum summation protocols based on single-photon states. By optimizing the communication topology and protocol structure, their schemes further reduce the dependence of classical users on quantum measurement capabilities. Early SQSMS studies mainly focused on two-party or three-party modulo-2 summation scenarios. More recently, research has gradually extended to arbitrary multi-party and modulo-*d* computation settings. High-dimensional quantum systems have attracted increasing attention in quantum information processing because they can encode more information per carrier and thus provide higher channel capacity and transmission efficiency than qubit-based systems. These advantages have led to a variety of high-dimensional quantum computation and communication schemes [31,32]. In 2024, Ye et al. [33] proposed a multi-party modulo-*d* summation protocol combining high-dimensional entangled states with single-particle states, enabling secure summation among arbitrary numbers of participants. However, the protocol requires massive high-dimensional quantum resources, resulting in exponentially increasing overhead. Meanwhile, Lian et al. [34] proposed a hybrid semi-quantum summation protocol supporting comparison, multiplication, and summation functions. Although the scheme improves resource utilization efficiency, it relies on a dual-trusted-party architecture, where collusion or deviation between trusted entities may compromise overall security.

Overall, although semi-quantum models significantly reduce the quantum capability requirements compared with fully quantum schemes, existing SQSMS protocols still suffer from several limitations, including dependence on pre-shared keys, reliance on multiple trusted parties, and relatively low quantum efficiency. Therefore, improving quantum resource utilization, reducing trust assumptions, and enhancing scalability while maintaining strict security guarantees remain important and challenging research problems.

To address these challenges, this paper proposes an efficient Semi-Quantum Secure Multi-party Summation protocol based on *d*-dimensional *n*-particle entangled states. The main contributions of this paper are summarized as follows:

- We propose an efficient Semi-Quantum Secure Multi-party Summation protocol based on *d*-dimensional *n*-particle entangled states. By utilizing the global correlations of entangled states to construct mutually cancelable random masks, the protocol achieves secure aggregation of private inputs while reducing quantum communication overhead.
- We analyze the correctness, security, and efficiency of the proposed protocol, and implement the corresponding quantum circuit model on the IBM Qiskit platform. Our theoretical analysis and simulation results verify the correctness, security, and feasibility of the protocol.
- Based on the proposed protocol, we further construct semi-quantum anonymous voting, anonymous auction, and anonymous ranking protocols, demonstrating its scalability and applicability in privacy-preserving multi-party computation scenarios.

The remainder of this paper is organized as follows. Section 2 introduces the preliminary knowledge used in this work, including basic mathematical notations, measurement bases, and related properties. Section 3 presents the detailed implementation process of the proposed protocol. Section 4 analyzes the correctness, security, and quantum efficiency of the protocol and evaluates its feasibility through simulation experiments. Section 5 discusses the extended anonymous application protocols. Finally, Section 6 concludes the paper and outlines future research directions.

## 2. Preliminary

### 2.1. Definition of Notations

Table 1 lists the definition of notations that will be used in our paper.

### 2.2. Two Measurement Bases

In high-dimensional quantum systems, the most commonly adopted measurement bases include the computational basis and the Fourier basis. Both play essential roles in quantum information processing and quantum communication protocols. In particular, for the construction and measurement analysis of high-dimensional entangled states, the transformation relationship between these two bases is generally established via the Quantum Fourier Transform (QFT).

For a *d*-dimensional quantum system, the computational basis is defined as

(1) $$B_{1}=\{|0\rangle ,|1\rangle ,\ldots ,|d-1\rangle \},$$

which forms a set of standard orthonormal bases of the *d*-dimensional Hilbert space. When a quantum state is measured under the computational basis it collapses into one of the basis states and the measurement outcome corresponds to the label of the collapsed basis state.

Correspondingly, the Fourier basis can be derived by applying the QFT to the computational basis. Let *F* denote the *d*-dimensional Quantum Fourier Transform operator; the Fourier basis is then defined as

(2) $$B_{2}=\{F|0\rangle ,F|1\rangle ,\ldots ,F|d-1\rangle \}.$$

The action of QFT on an arbitrary computational basis state |j〉 is given by

(3) $$F|j\rangle =\frac{1}{\sqrt{d}}\sum_{k=0}^{d-1}e^{\frac{2\pi ijk}{d}}|k\rangle .$$

Accordingly, each basis state in the Fourier basis is a uniform superposition of computational basis states with specific phase factors. A one-to-one correspondence between the computational basis and the Fourier basis is established via QFT and its inverse transform ${\mathrm{QFT}}^{†}$, making them a pair of mutually unbiased bases.

High-dimensional entangled states possess distinctive nonlocal correlation properties under different measurement bases. The mathematical expression of the *d*-dimensional *n*-particle GHZ entangled state is given by

(4) $$|\Phi \rangle =\frac{1}{\sqrt{d}}\sum_{j=0}^{d-1}{|j\rangle }_{1}{|j\rangle }_{2}\cdots {|j\rangle }_{n},$$

Such high-dimensional GHZ entangled states have remarkable particle-to-particle nonlocal correlations. This section introduces complementary correlation characteristics of the original state and its Fourier-transformed counterpart under two typical measurement bases. Note that the detailed derivations of all equations involved in the following properties are available in Refs. [35,36]. The relevant inherent properties are listed below.

**Property 1.**  *For |Φ〉, if all particles are measured under the computational basis $B_{1}$ then the resulting measurement outcomes are perfectly correlated. Specifically, all participants obtain the same measurement result, namely*

(5) $$m_{1}=m_{2}=\cdots =m_{n}.$$

**Property 2.**  *For |Φ〉, if all particles are measured under the Fourier basis $B_{2}$ then the measurement outcomes satisfy a modular summation constraint such that*

(6) $$m_{1}+m_{2}+\cdots +m_{n}\equiv 0\;\left(\mathrm{mod}\;d\right).$$

*Furthermore, by applying the Quantum Fourier Transform F to each particle of |Φ〉, the following quantum state can be obtained:*

(7) $$|\Psi \rangle =\frac{1}{\sqrt{d}}\sum_{j=0}^{d-1}{F|j\rangle }_{1}⊗{F|j\rangle }_{2}⊗\cdots ⊗F{|j\rangle }_{n}.$$

*The transformed state |Ψ〉 can be regarded as the Fourier-domain representation of |Φ〉, and it exhibits complementary correlation properties under the two measurement bases.*

**Property 3.**  *For |Ψ〉, if all particles are measured under the computational basis $B_{1}$ then the measurement outcomes satisfy the following modular relation:*

(8) $$m_{1}+m_{2}+\cdots +m_{n}\equiv 0\;\left(\mathrm{mod}\;d\right).$$

**Property 4.**  *For |Ψ〉, if all particles are measured under the Fourier basis $B_{2}$ then the resulting measurement outcomes are completely identical, i.e.,*

(9) $$m_{1}=m_{2}=\cdots =m_{n}.$$

## 3. Proposed Protocol

This protocol considers a computation model consisting of *n* mutually untrusted classical participants $P_{1},P_{2},\ldots ,P_{n}$ and one quantum participant TP. Each classical participant $P_{i}$ holds a private input $x_{i}\in \left\{0,1,\ldots ,d-1\right\}$, where *d* denotes the dimension of the quantum system and the public modulo parameter. The objective is to collaboratively compute the summation result $R=\sum_{i=1}^{n}x_{i}\;\left(\mathrm{mod}\;d\right)$ without revealing any participant’s private information. In the security model, all classical participants are mutually untrusted and may attempt to infer others’ private inputs from the exchanged information, while still following the protocol honestly. As a remote quantum server, TP is assumed to be semi-honest, i.e., it faithfully executes the protocol but may analyze the obtained information. Moreover, TP is not allowed to collude with any participant or tamper with the protocol execution.

**Protocol 1.**  *Semi-Quantum Secure Multi-Party Summation Protocol (SQSMS).*

**Input:** Each classical participant $P_{i}$(i=1,2,…,n) holds a private input $x_{i}\in \left\{0,1,\ldots ,d-1\right\}$.

**Output:** Compute the summation result $R=\sum_{i=1}^{n}x_{i}\;\left(\mathrm{mod}\;d\right)$ without revealing any participant’s private input.

**(1)**  **Initialization Phase**

Step 1. Each classical participant $P_{i}$ constructs a binary encoding sequence $X_{i}=\left\{x_{i0},x_{i1},\ldots ,x_{i(d-1)}\right\}$ of length *d*, where $x_{ij}\in \left\{0,1\right\}$ for j∈{0,1,…,d−1}. The encoding rule is defined as:

(10) $$x_{ij}=\left\{\begin{array}{cc} 1 & \mathrm{if}\;j=x_{i},\\ 0 & \mathrm{if}\;j\neq x_{i}. \end{array}\right.$$

Step 2. TP prepares *d* sets of the *d*-dimensional *n*-particle GHZ entangled state defined previously, which takes the form

(11) $$|\Phi \rangle =\frac{1}{\sqrt{d}}\sum_{j=0}^{d-1}{\left(|j\rangle \right)}^{⊗n}=\frac{1}{\sqrt{d}}\sum_{j=0}^{d-1}{|j\rangle }_{1}{|j\rangle }_{2}\cdots {|j\rangle }_{n}.$$

TP then applies the QFT to every particle of this state, and the resulting state is written as

(12) $$|\psi \rangle =\frac{1}{\sqrt{d}}\sum_{j=0}^{d-1}{\left(F|j\rangle \right)}^{⊗n}=\frac{1}{\sqrt{d}}\sum_{j=0}^{d-1}{F|j\rangle }_{1}{F|j\rangle }_{2}\cdots F{|j\rangle }_{n},$$

where *F* denotes the *d*-dimensional QFT operator. TP subsequently partitions all entangled states according to particle positions. The *i*-th quantum sequence $S_{i}$ consists of the *i*-th particle selected from all prepared states, forming the sequence set $S_{1},S_{2},\ldots ,S_{n}$.

Step 3. TP additionally prepares δ sets of identical original entangled states |Φ〉 as decoy states. Half of these decoy state sets are chosen randomly, and the predefined operator *F* is applied to all particles to generate transformed decoy states |ψ〉. Following the same particle partitioning operation described in Step 2, TP divides all δ decoy state sets into *n* groups and randomly inserts each group into the corresponding quantum sequence $S_{i}$. The updated sequences are marked as $S_{1}^{\prime },S_{2}^{\prime },\ldots ,S_{n}^{\prime }$, and TP records all insertion positions for subsequent eavesdropping detection.

**(2)**  **Interactive Phase**

Step 4. After each classical participant $P_{i}$ (i=1,2,…,n) receives the corresponding quantum sequence $S_{i}^{\prime }$, TP publicly announces the positions of all decoy particles together with the corresponding measurement bases. According to the published information, each participant locates the decoy particles and performs measurements using the specified bases. The measurement results are then returned to TP for consistency verification.

To verify the security of the quantum channel, TP checks the consistency of all reported measurement results according to the preparation method of the decoy particles and the selected measurement bases. The corresponding criteria are listed in Table 2.

TP calculates the error rate according to the above criteria. If the error rate is below the preset threshold then the quantum channel is regarded as secure and the protocol continues. Otherwise, the protocol is terminated due to possible eavesdropping.

Step 5. After the eavesdropping detection is completed, each participant removes all decoy particles from the received sequence. Participant $P_{i}$ then sequentially measures the remaining quantum particles and records the corresponding outcomes $r_{ij}$, where j∈{0,1,…,d−1} denotes the *j*-th entangled state.

**Figure 1:**
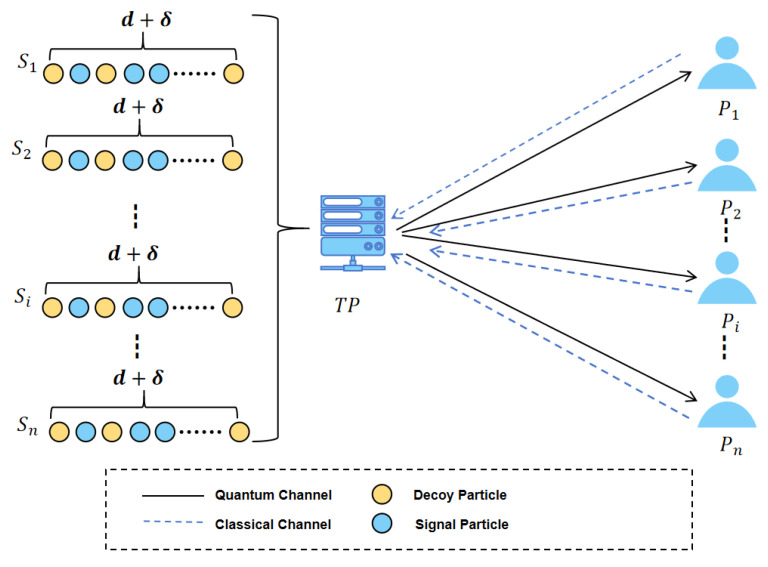
Schematic diagram of interactive phase operation.

Step 6. Each participant $P_{i}$ encrypts the private sequence $X_{i}$ using the measurement results $r_{ij}$. The encryption rule is defined as

(13) $$c_{ij}=x_{ij}+r_{ij}\;\left(\mathrm{mod}\;d\right),$$

where i∈{1,…,n}, j∈{0,…,d−1}, and where $x_{ij}$ represents the *j*-th element of $X_{i}$. Each participant constructs the encrypted sequence $X_{i}^{\prime }=\left(c_{i0},c_{i1},\ldots ,c_{i(d-1)}\right)$ and sends it to TP. The interaction process is illustrated in Figure 1.

**(3)**  **Output Result Phase**

Step 7. After receiving all the encrypted sequences, TP performs the following summation for each index *j*:

(14) $$f_{j}=\sum_{i=1}^{n}c_{ij}=\sum_{i=1}^{n}\left(x_{ij}+r_{ij}\right)=\sum_{i=1}^{n}x_{ij}.$$

Finally, TP computes the output result by

(15) $$R=\sum_{j=0}^{d-1}j\cdot f_{j}\;\left(\mathrm{mod}\;d\right).$$

## 4. Performance Analysis

This section presents a comprehensive performance evaluation of the SQSMS protocol, including its correctness, security, efficiency, and practical feasibility. The analysis is conducted through theoretical derivation, comparative assessment, and simulation verification to demonstrate the protocol’s reliability and applicability in realistic scenarios.

### 4.1. Correctness

This section proves that the proposed SQSMS protocol can correctly compute the following result when all participants honestly execute the protocol:

(16) $$R=\sum_{i=1}^{n}x_{i}\;\left(\mathrm{mod}\;d\right).$$

In the initialization phase, each participant $P_{i}$ encodes its private input $x_{i}\in \left\{0,1,\ldots ,d-1\right\}$ into a binary sequence $X_{i}=\left\{x_{i0},x_{i1},\ldots ,x_{i(d-1)}\right\}$ of length *d*, where

(17) $$x_{ij}=\left\{\begin{array}{cc} 1, & j=x_{i},\\ 0, & j\neq x_{i}, \end{array}\right.\;j\in \left\{0,1,\ldots ,d-1\right\}.$$

Accordingly, each encoding sequence satisfies

(18) $$\sum_{j=0}^{d-1}x_{ij}=1.$$

Summing over all participants gives

(19) $$\sum_{j=0}^{d-1}j\left(\sum_{i=1}^{n}x_{ij}\right)=\sum_{i=1}^{n}x_{i}.$$

In the SQSMS protocol, TP first prepares the original d-dimensional n-particle entangled states. It then applies the operator *F* to all particles of the entangled states before distributing them to each participant. As stated in Property 3 of the preliminary section, the transformed entangled states retain their inherent correlation constraints. Accordingly, the measurement results $r_{ij}$ obtained by participants from the received quantum states satisfy

(20) $$\sum_{i=1}^{n}r_{ij}\equiv 0\;\left(\mathrm{mod}\;d\right).$$

Each participant encrypts the private encoding sequence according to Equations (13) and (14). TP then computes the aggregation value at the *j*-th position:

(21) $$f_{j}=\sum_{i=1}^{n}c_{ij}=\sum_{i=1}^{n}x_{ij}+\sum_{i=1}^{n}r_{ij}\equiv \sum_{i=1}^{n}x_{ij}.$$

Therefore, the masking terms are eliminated during aggregation and do not affect the correctness of the summation result.

In the output phase, TP reconstructs the final result according to Equations (13)–(15). Since $f_{j}=\sum_{i=1}^{n}x_{ij},$ we have

(22) $$R=\sum_{j=0}^{d-1}j\cdot f_{j}\;\left(\mathrm{mod}\;d\right)=\sum_{i=1}^{n}x_{i}\;\left(\mathrm{mod}\;d\right).$$

Therefore, the protocol can correctly compute the summation of all private inputs under honest conditions.

To further illustrate the correctness of the protocol, consider a simple example with n=3 and d=5. Suppose the private inputs are $x_{1}=1$, $x_{2}=3$, and $x_{3}=4$. According to the one-hot encoding rule, the corresponding encoding sequences are (0,1,0,0,0),(0,0,0,1,0),(0,0,0,0,1).

Bitwise aggregation yields $f_{0}=0,\;f_{1}=1,\;f_{2}=0,\;f_{3}=1,\;f_{4}=1.$

During the quantum measurement phase, the entangled correlation guarantees that the masking terms satisfy

$$\sum_{i=1}^{n}r_{ij}\equiv 0\;\left(\mathrm{mod}\;d\right).$$

Hence,

$$f_{j}=\sum_{i=1}^{3}x_{ij}.$$

Substituting the aggregation results into the reconstruction formula gives

(23) R=0×0+1×1+2×0+3×1+4×1=8≡3(mod5).

The direct summation of the original private inputs is

1+3+4=8≡3(mod5).

The two results are identical, which verifies the correctness of the proposed protocol.

### 4.2. Security

This section analyzes the security of the proposed SQSMS protocol against six representative attack models, namely measurement attack, intercept–resend attack, entangled measurement attack, impersonation attack, collusion attack, and external attack. The security analysis is conducted under the semi-honest model, where all participants follow the protocol execution while potentially attempting to infer additional information from the accessible data.

**Lemma 1.** 
*Resistance to measurement attack. Any adversary performing unauthorized measurements on the transmitted quantum sequences can be detected during the eavesdropping detection process.*

**Proof.**  Suppose an adversary intercepts a transmitted sequence and performs single-particle measurements before forwarding the particles.

According to Step 3, TP randomly inserts δ decoy entangled states into each transmitted sequence. Some decoy states remain in the original GHZ state |Φ〉, while the others are transformed into |ψ〉 by applying QFT.

After receiving the particles, participants measure the decoy particles according to the measurement bases announced by TP.

For the original GHZ state |Φ〉, Property 1 shows that all measurement outcomes must be identical in the computational basis, while Property 2 requires

(24) $$\sum_{i=1}^{n}r_{ij}\equiv 0\;\left(\mathrm{mod}\;d\right)$$

in the Fourier basis.

Similarly, for the transformed state |ψ〉 the two correlation conditions are exchanged.

Since the adversary does not know the positions of decoy particles or whether QFT has been applied, any unauthorized measurement collapses the corresponding quantum state and disturbs the original correlations.

Consequently, the reported measurement outcomes no longer satisfy the verification criteria listed in Table 2, leading to an abnormal error rate. Therefore, the attack is detected by TP during Step 4. □

**Lemma 2.** 
*Resistance to intercept–resend attack. The proposed SQSMS protocol can resist intercept–resend attacks.*

**Proof.**  Suppose an adversary intercepts a transmitted sequence, measures the intercepted particles, prepares new particles according to the measurement outcomes, and then resends them to the legitimate participant.

Because the adversary has no knowledge of the locations of the decoy particles and does not know whether a decoy state belongs to |Φ〉 or |ψ〉 the reconstructed particles cannot preserve the original correlation structure.

Consequently, the forged particles fail to satisfy the correlation conditions required by Table 2. During the eavesdropping detection process, TP compares all reported measurement outcomes and computes the error rate.

Therefore, the intercept–resend attack inevitably introduces detectable inconsistencies and is discovered before the protocol proceeds to the summation stage. □

**Lemma 3.** 
*Resistance to collusion attack. Assuming that TP does not collude with any participant, any subset of dishonest participants cannot recover the private inputs of honest participants.*

**Proof.**  Suppose a coalition $C=\{P_{1},P_{2},\ldots ,P_{k}\}$ attempts to infer the private input of an honest participant $P_{t}$, where k<n−1.

According to Step 6, participant $P_{t}$ uploads

(25) $$c_{tj}=x_{tj}+r_{tj}\;\left(\mathrm{mod}\;d\right).$$

The masking value $r_{tj}$ originates from the measurement outcomes of the shared high-dimensional entangled state and satisfies

(26) $$\sum_{i=1}^{n}r_{ij}\equiv 0\;\left(\mathrm{mod}\;d\right).$$

Although the colluding participants know their own measurement results they cannot obtain the measurement outcomes belonging to honest participants. Therefore, they cannot determine the exact value of $r_{tj}$.

Since $r_{tj}$ remains unknown to the colluding set, the ciphertext $c_{tj}$ reveals no useful information about $x_{tj}$. Consequently, the coalition cannot recover the private input of the honest participant. □

**Lemma 4.** 
*Resistance to external attack. External adversaries cannot obtain valid information through either the quantum channel or the classical channel.*

**Proof.**  For the quantum channel, an external adversary may perform measurement attacks, intercept–resend attacks, or entangled measurement attacks by introducing auxiliary particles and performing joint operations.

However, any operation on the transmitted particles disturbs the correlations of the original entangled states. As a result, the measurement outcomes of the decoy particles no longer satisfy the verification criteria in Table 2, and the attack is detected during Step 4.

For the classical channel, the transmitted information is

(27) $$c_{ij}=x_{ij}+r_{ij}\;\left(\mathrm{mod}\;d\right).$$

Since the masking value $r_{ij}$ is generated from the quantum measurement outcomes and is unavailable to external adversaries, the ciphertext sequence alone is insufficient to recover any participant’s private input.

Therefore, external attacks cannot obtain useful information from either communication channel. □

### 4.3. Efficiency Evaluation

For this section, we systematically evaluated the performance of the proposed SQSMS protocol. Several metrics were adopted for comparison with existing quantum summation protocols, including quantum resource consumption, summation type, quantum operation complexity, reliance on pre-shared keys, the number of TPs, and quantum efficiency. The detailed comparison results are presented in Table 3.

In a *d*-dimensional Hilbert space, quantum efficiency is widely used to evaluate the communication efficiency of a quantum protocol, which is defined as

(28) $$\eta =\frac{c}{q+b},$$

where *c* denotes the total length of encoded plaintext information involved in the computation, *q* represents the total number of consumed *d*-dimensional quantum particles (i.e., qudits), and *b* denotes the total number of classical bits exchanged for decoding.

Consider the SQSMS protocol with *n* participants, where each participant encodes one private input into a sequence of length *d*, and δ decoy particles are employed for channel security detection. During protocol execution, TP prepares *d* *d*-dimensional *n*-particle entangled states for information transmission and additional δ entangled states as decoy states. Therefore, the total number of consumed quantum particles is

(29) q=n(d+δ).

In Step 6, each participant sends an encrypted sequence $X_{i}^{\prime }=\left(c_{i0},c_{i1},\ldots ,c_{i(d-1)}\right)$ of length *d* to TP. According to the encoding procedure in Step 1, each private input $x_{i}$ is first mapped into a binary sequence $X_{i}=\left(x_{i0},x_{i1},\ldots ,x_{i(d-1)}\right)$ of length *d*. Since the subsequent encryption, transmission, and summation operations are all performed on these encoded elements, the total amount of plaintext information involved in the computation is nd. Therefore, the total length of plaintext messages is c=nd.

Accordingly, the quantum communication efficiency of the proposed SQSMS protocol is

(30) $$\eta =\frac{nd}{n(d+\delta )+nd}=\frac{d}{2d+\delta }.$$

It can be observed that for a fixed number of participants and security parameter δ the quantum efficiency gradually approaches a constant as the encoding dimension *d* increases:

(31) $$\lim_{d\to \infty }\eta =\frac{1}{2}.$$

Therefore, while guaranteeing channel security detection the proposed protocol achieves linear-scale resource consumption and maintains stable quantum communication efficiency for large-scale inputs.

As shown in Table 3, the proposed SQSMS protocol exhibits several advantages in key performance indicators. In terms of quantum resources, the protocol adopts *d*-dimensional *n*-particle entangled states as information carriers and completes the summation task using only measurement operations, without introducing complex quantum logic gates such as SUM gates or $U_{k}$ gates. Therefore, the protocol has lower implementation complexity than the scheme in Ref. [25]. Although the protocol in Ref. [34] employs *d*-dimensional single-particle states it requires two TPs, resulting in higher system complexity. By contrast, the proposed protocol relies only on a single semi-honest TP and does not require pre-shared keys, thereby avoiding additional overhead caused by key distribution and management. In terms of functionality, the proposed protocol supports modulo-*d* summation, making it more suitable for high-dimensional data processing than modulo-2 summation schemes. Quantitatively, the protocol achieves quantum efficiency $\frac{d}{2d+\delta }$, which is higher than most existing modulo-*d* summation protocols.

Overall, the proposed SQSMS protocol achieves favorable performance in terms of quantum operation complexity, system structure simplicity, trust model rationality, and communication scalability, while maintaining satisfactory security guarantees.

### 4.4. Simulation

Numerical simulations were conducted using the IBM Qiskit platform to verify the logical correctness and internal consistency of the proposed SQSMS protocol under ideal quantum circuit settings. In the considered model, the trusted third party (TP) prepares high-dimensional GHZ entangled states as quantum resources and distributes them to the participants. Each participant performs measurements in either the computational basis or the Fourier basis to generate correlated outcomes and to verify consistency relations required by the protocol. It is important to emphasize that the simulation was performed on an ideal noise-free quantum circuit model and was used solely to validate the theoretical measurement correlations and protocol functionality, rather than to evaluate physical implementation performance on near-term quantum hardware.

Without loss of generality, an 8-dimensional three-particle quantum system was considered for simulation. The TP prepared the following two groups of entangled states:

(32) $$|\Phi \rangle =\frac{1}{\sqrt{8}}\sum_{j=0}^{7}{|j\rangle }_{1}{|j\rangle }_{2}{|j\rangle }_{3},$$

(33) $$|\psi \rangle =\frac{1}{\sqrt{8}}\sum_{j=0}^{7}{F|j\rangle }_{1}{F|j\rangle }_{2}F{|j\rangle }_{3}.$$

**Figure 2:**
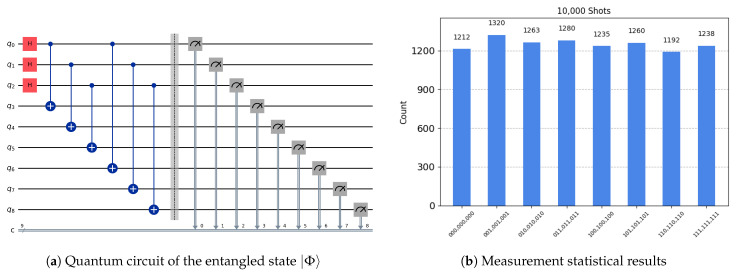
Quantum circuit and computational basis measurement results of the entangled state |Φ〉.

When measuring the original entangled state |Φ〉 via the computational basis, three participants obtained identical measurement outcomes in each trial. The measured results were uniformly distributed over {000,001,010,011,100,101,110,111}. The corresponding quantum circuit and measurement results are displayed in Figure 2.

**Figure 3:**
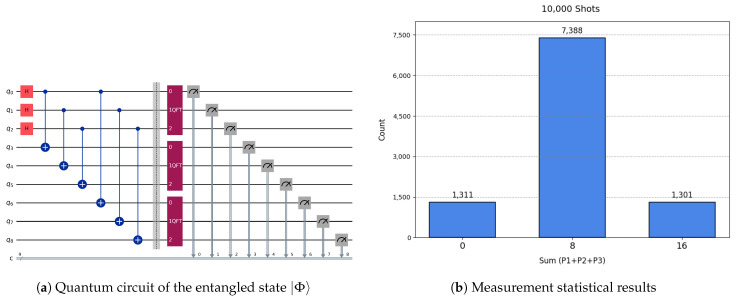
Quantum circuit and Fourier basis measurement results of the entangled state |Φ〉.

When adopting the Fourier basis to measure |Φ〉, the sum of three groups of measurement results was concentrated on 0, 8 and 16, which complied well with the theoretical modulo-*d* constraint. The relevant simulation results are illustrated in Figure 3.

**Figure 4:**
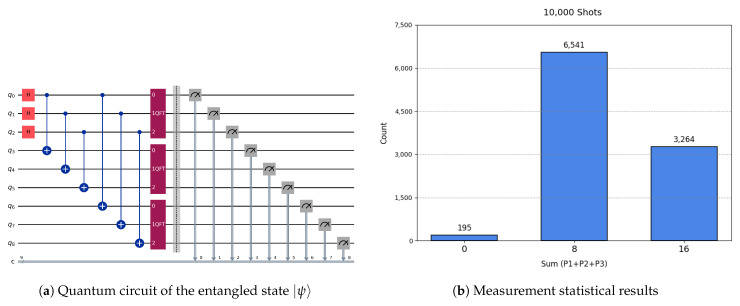
Quantum circuit and computational basis measurement results of the entangled state |ψ〉.

For the Fourier-transformed entangled state |ψ〉, the computational basis measurement results satisfied the constraint ∑≡0(modd), and the summation values were concentrated on 0, 8 and 16. The corresponding quantum circuit and result distribution are shown in Figure 4.

**Figure 5:**
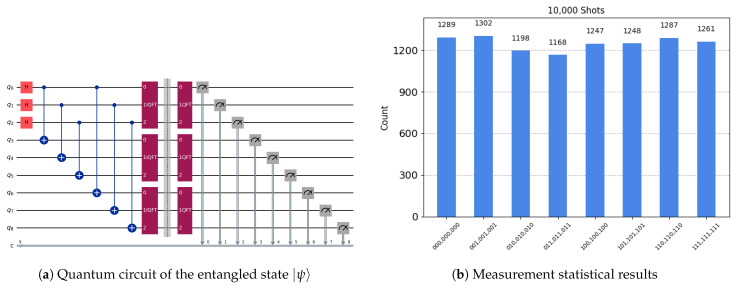
Quantum circuit and Fourier basis measurement results of the entangled state |ψ〉.

By contrast, the Fourier basis measurements on |ψ〉 generated consistent outcomes among the three participants in each simulation. All the measurement results followed a uniform distribution across {000,001,010,011,100,101,110,111}. The quantum circuit and statistical results are presented in Figure 5. Overall, the Qiskit simulations confirmed the expected entanglement correlations and measurement consistency of the proposed protocol under ideal quantum circuit assumptions.

## 5. Applications

In this section, we further extend the proposed SQSMS protocol and design three types of semi-quantum multi-party computation protocols suitable for anonymous scenarios, namely anonymous voting, anonymous auction, and anonymous ranking. Since these three protocols are constructed based on the SQSMS framework, we do not repeat the specific implementation steps of the original protocol. Instead, we focus on analyzing how to adopt the SQSMS protocol to construct the anonymous voting, anonymous auction, and anonymous ranking protocols.

### 5.1. Semi-Quantum Anonymous Voting Protocol

**Definition 1** 
(Semi-quantum anonymous voting). *Suppose there exists a trusted quantum-assisted third party TP and n voters $\left\{P_{1},P_{2},\ldots ,P_{n}\right\}$. Each voter $P_{i}$ holds a private vote $x_{i}\in \left[0,d-1\right]$. The goal is to count the candidate with the highest number of votes while ensuring the anonymity of both voting contents and voter identities.*

**Protocol 2.**  *Semi-quantum Secure Multi-party Anonymous Voting Protocol (SQSMAV)*

**Input:** Each participant $P_{i}$ inputs a private vote $x_{i}$.

**Output:** The TP announces the candidate number with the maximum number of votes.

**Step 1. Parameter publication.** The TP releases the candidate set {0,1,…,d−1} and the maximum number of participants *n*.

**Step 2. Voting vector generation.** Each voter $P_{i}$ determines its private vote $x_{i}\in \left[0,d-1\right]$ and constructs a voting vector $v_{i}$ of length *d*. The $x_{i}$-th entry is set to 1, and all other entries are set to 0.

(34) $$R=\left(r_{0},\ldots ,r_{l},\ldots ,r_{d}-1\right)=\left(\begin{array}{c} r_{10}\\ \vdots \\ r_{i0}\\ \vdots \\ r_{n0} \end{array}\right)\cdots \left(\begin{array}{c} r_{1l}\\ \vdots \\ r_{il}\\ \vdots \\ r_{nl} \end{array}\right)\cdots \left(\begin{array}{c} r_{1d-1}\\ \vdots \\ r_{id-1}\\ \vdots \\ r_{nd-1} \end{array}\right).$$

**Step 3. Mask generation.** All voters generate shared masks $r_{i}$ through the SQSMS protocol.

**Step 4. Encryption and transmission.** Each $P_{i}$ encrypts its voting vector with the generated mask:

(35) $$v_{il}^{*}=v_{il}+r_{il},\;l=0,1,\ldots ,d-1.$$

Then each participant sends the encrypted vector to the TP.

**Step 5. Vote counting and statistics.** The TP initializes the vote vector $W=\{w_{0},w_{1},\ldots ,w_{d}-1\}$ and calculates

(36) $$w_{l}=\sum_{i=1}^{n}\left(v_{il}+r_{il}\right)\;\mathrm{mod}\;d,\;l=0,1,\ldots ,d-1.$$

Finally, the TP publishes the statistical result *W* to all voters and announces the candidate with the highest votes.

**Example 1.** 
*To illustrate the execution of the SQSMAV protocol, a concrete instance is given as follows. Suppose there exists a trusted quantum-assisted third party TP with n=3 voters $\left\{P_{1},P_{2},P_{3}\right\}$, and the number of candidates is d=3 indexed by {0,1,2}.*

*First, the TP releases the candidate set and the maximum number of participants. Subsequently, the three voters cast their votes independently: $P_{1}$ and $P_{3}$ vote for Candidate 1, while $P_{2}$ votes for Candidate 2, i.e., $x_{1}=0$, $x_{2}=1$, $x_{3}=0$. According to the protocol, each voter constructs a voting vector of length 3: $v_{1}=\left(1,0,0\right)$, $v_{2}=\left(0,1,0\right)$, and $v_{3}=\left(1,0,0\right)$.*

*Then, the three voters generate a shared random mask matrix $R=(r_{il})$ via the SQSMS protocol, satisfying*

(37) $$\sum_{i=1}^{3}r_{il}\equiv 0\;\left(\mathrm{mod}\;10\right).$$

*Assume the generated mask matrix is*

$$R=\left(\begin{array}{ccc} 2 & 4 & 3\\ 5 & 1 & 6\\ 3 & 5 & 1 \end{array}\right).$$

Each voter encrypts its voting vector by $v_{il}^{*}=\left(v_{il}+r_{il}\right)\;\mathrm{mod}\;10$, yielding the encrypted voting vectors $v_{1}^{*}=\left(3,4,3\right)$, $v_{2}^{*}=\left(5,2,6\right)$, and $v_{3}^{*}=\left(4,5,1\right)$, which are then transmitted to the TP.

*After receiving all the encrypted vectors, the TP initializes the vote vector $W=(w_{0},w_{1},w_{2})$, computes*

$$w_{l}=\sum_{i=1}^{3}\left(v_{il}+r_{il}\right)\;\mathrm{mod}\;10,$$

*and obtains W=(2,1,0). Since the column sum of masks is zero modulo d, the random masks cancel out automatically during aggregation, and the statistical result coincides with the real vote count. It follows that Candidate 0 obtains 2 votes, Candidate 1 obtains 1 vote, and Candidate 2 obtains 0 votes.*

*This example demonstrates that the TP only obtains encrypted data throughout the process and cannot infer the specific vote of any voter. The protocol achieves anonymity of voting content and voter identity while guaranteeing the correctness of statistical results.*

**Correctness.** The correctness of the SQSMAV protocol can be directly derived from the correctness of the SQSMS protocol. Each voter encodes its vote into a unit vector, and all participants generate shared masks satisfying

(38) $$\sum_{i=1}^{n}r_{ij}\equiv 0\;\left(\mathrm{mod}\;d\right)$$

via the SQSMS protocol. When the TP performs bitwise aggregation, the random mask terms in the encrypted data cancel each other out modulo *d*, yielding the true statistical result

$$w_{l}=\sum_{i=1}^{n}v_{il}.$$

Accordingly, the TP can accurately count the votes of each candidate and correctly determine the winner with the highest votes. Therefore, the protocol guarantees the correctness of voting statistics under the honest execution of all participants.

**Security.** The SQSMAV protocol inherits the information-theoretic security of the SQSMS protocol. Since each mask $r_{il}$ obeys a uniform distribution modulo *d*, the encrypted data is statistically independent of the original vote value. Thus, neither the TP nor external eavesdroppers can recover the private vote of any voter. Meanwhile, all voters submit their vectors anonymously; the TP only obtains the aggregated result and cannot establish the correspondence between voting content and voter identity, realizing identity anonymity. Under the semi-honest and non-full-collusion model, any subset of participants cannot eliminate the uncertainty introduced by unknown masks. Hence, the protocol can effectively resist collusion attacks and preserve voting privacy.

### 5.2. Semi-Quantum Anonymous Auction Protocol

**Definition 2** 
(Semi-quantum anonymous auction). *Suppose there exists a semi-honest auctioneer TP and n bidders $\left\{P_{1},P_{2},\ldots ,P_{n}\right\}$. Each bidder $P_{i}$ holds a private bid $x_{i}\in \left[0,N-1\right]$. The objective is to determine the unique highest bidder while preserving bid privacy and identity anonymity.*

**Protocol 3.**  *Semi-Quantum Secure Multi-party Anonymous Auction Protocol (SQSMAA).*

**Input:** Each bidder $P_{i}$ submits its private bid $x_{i}$.

**Output:** The TP announces the unique winner without disclosing any bid values.

**Step 1. Parameter negotiation.** The TP and bidders agree on two public parameters: subinterval width Δ and the number of intervals L=⌈N/Δ⌉. The whole range is divided into *L* consecutive subintervals: [0,Δ),[Δ,2Δ),…,[N−Δ,N). The modulus parameter satisfies d>n.

**Step 2. Vector mapping.** Each bidder $P_{i}$ constructs an *L*-length vector $v_{i}$. If $x_{i}\in \left[l\Delta ,\left(l+1\right)\Delta \right)$ then set $v_{il}=1$ and 0 otherwise.

**Step 3. Mask generation and encryption.** All bidders execute the SQSMS protocol to generate the shared mask matrix *R* as shown in Equation (34). Each bidder encrypts its vector bitwise:

(39) $$v_{il}^{*}=v_{il}+r_{il},\;l=0,1,\ldots ,L-1.$$

The encrypted vector $v_{i}^{*}$ is sent to the TP via a classical channel.

**Step 4. Preliminary judgment.** The TP performs the following for l=0,1,…,L−1:

- If $\sum_{i=1}^{n}\left(v_{il}+r_{il}\right)\;\mathrm{mod}\;d=0$ then no bid in this interval: continue;
- If $\sum_{i=1}^{n}\left(v_{il}+r_{il}\right)\;\mathrm{mod}\;d>1$ then multiple bidders exist: go to Step 5;
- If $\sum_{i=1}^{n}\left(v_{il}+r_{il}\right)\;\mathrm{mod}\;d=1$ then unique bidder found: go to Step 6.

**Step 5. Fine-grained iteration.** If multiple bidders exist in the current interval, reduce Δ, repartition the range, and repeat Steps 1–4 until the unique highest bidder is found.

**Step 6. Winner announcement.** If only one bidder exists in the current interval then the TP announces the winner without revealing any bid values or private information.

**Example 2.** 
*To illustrate the execution of the SQSMAA protocol we provide a concrete example. Consider a semi-honest auctioneer TP and n=3 bidders $\left\{P_{1},P_{2},P_{3}\right\}$ with a bid range [0,15] (N=16). Their private bids are $x_{1}=5$, $x_{2}=12$, and $x_{3}=9$.*

*First, the TP and bidders set Δ=4 and L=⌈16/4⌉=4. The range is divided into [0,4),[4,8),[8,12),[12,16). The bidders construct vectors: $v_{1}=\left(0,1,0,0\right)$, $v_{2}=\left(0,0,0,1\right)$, $v_{3}=\left(0,0,1,0\right)$.*

*They generate a shared mask matrix $R=(r_{il})$ via SQSMS, satisfying*

(40) $$\sum_{i=1}^{3}r_{il}\equiv 0\;\left(\mathrm{mod}\;d\right).$$

*Let d=10 and let the mask matrix be*

$$R=\left(\begin{array}{cccc} 3 & 2 & 4 & 1\\ 5 & 3 & 2 & 6\\ 2 & 5 & 4 & 3 \end{array}\right).$$

Each bidder encrypts its vector by $v_{il}^{*}=\left(v_{il}+r_{il}\right)\;\mathrm{mod}\;10$, obtaining $v_{1}^{*}=\left(3,3,4,1\right)$, $v_{2}^{*}=\left(5,3,2,7\right)$, $v_{3}^{*}=\left(2,5,5,3\right)$.

*The TP calculates*

$$\sum_{i=1}^{3}\left(v_{il}+r_{il}\right)\;\mathrm{mod}\;10$$

*and obtains (0,1,1,1). Since masks cancel out, the result reflects the real distribution. The TP checks from the highest interval and finds that the fourth interval has a count of 1, so $P_{2}$ is the winner. The bid values and identities remain anonymous.*

**Correctness.** The correctness of SQSMAA follows directly from that of SQSMS. By mapping bids to interval vectors and encrypting with zero-sum masks the TP obtains

(41) $$\sum_{i=1}^{n}\left(v_{il}+r_{il}\right)\equiv \sum_{i=1}^{n}v_{il}\;\left(\mathrm{mod}\;d\right),\;l=0,1,\ldots ,L-1,$$

which accurately counts bidders in each interval. By checking from high to low and refining intervals the TP correctly identifies the unique highest bidder.

**Security.** All bid vectors are encrypted with uniformly random masks modulo *d*. The TP cannot recover any private bid and only obtains interval-level statistics. Only the winner’s identity is disclosed, and no exact bids are leaked. Masks in different rounds are independent, preventing correlation attacks. Under the semi-honest model, the protocol resists eavesdropping and limited collusion, ensuring bid privacy and identity anonymity.

### 5.3. Semi-Quantum Anonymous Sorting Protocol

**Definition 3** 
(Semi-quantum anonymous ranking). *Suppose there are n participants $\left\{U_{1},U_{2},\ldots ,U_{n}\right\}$ and a trusted third party TP. Each participant $U_{i}$ holds a private input $x_{i}\in \left[0,L-1\right]$. The protocol aims to anonymously compute the ascending or descending ranking position of each participant among all the data without disclosing any private input values and identity information.The modulus parameter satisfies d>n to avoid ambiguity in aggregation statistics.*

**Protocol 4.**  *Semi-Quantum Secure Multi-party Anonymous Ranking Protocol (SQSMAS).*

**Input:** Private integer $x_{i}$ of participant $U_{i}$.

**Output:** Each participant obtains its own ranking result, while it cannot infer the private inputs or rankings of others.

**Step 1. Private value encoding.** Each participant $U_{i}$ maps its private input $x_{i}$ into an *L*-dimensional vector $v_{i}$ defined as

(42) $$v_{i}=\left[v_{i0},v_{i1},\ldots ,v_{i(L-1)}\right],\;v_{ij}=\left\{\begin{array}{cc} 1, & x_{i}=j,\\ 0, & \mathrm{otherwise}. \end{array}\right.$$

**Step 2. Mask generation.** All participants negotiate and generate the mask set *R* via the SQSMS protocol, as shown in Equation (34). Each participant $U_{i}$ encrypts its vector bitwise with the generated mask:

(43) $$v_{il}^{*}=\left(v_{il}+r_{il}\right)\;\mathrm{mod}\;d,\;l=0,1,\ldots ,L-1.$$

The encrypted vector is denoted as $v_{i}^{*}$ and transmitted to the TP through the classical channel.

**Step 3. Encrypted aggregation.** The TP aggregates the vector data of all the participants bit by bit:

(44) $$w_{l}=\sum_{i=1}^{n}\left(v_{il}+r_{il}\right)\;\mathrm{mod}\;d=\left(\sum_{i=1}^{n}v_{il}\right)\;\mathrm{mod}\;d,\;l=0,1,\ldots ,L-1.$$

Since the sum of all the masks satisfies the zero-sum property modulo *d*, the correctness of the final statistical result is guaranteed.

**Step 4. Anonymous ranking publication.** The TP publishes the aggregated vector $W=[w_{0},w_{1},\ldots ,w_{L-1}]$ to all participants. Combined with its own private input $x_{i}$, each participant locally calculates its ascending and descending ranking:

Ascending ranking:

(45) $${\mathrm{Rank}}_{\mathrm{asc}}\left(x_{i}\right)=1+\sum_{l=0}^{x_{i}-1}w_{l}.$$

Descending ranking:

(46) $${\mathrm{Rank}}_{\mathrm{desc}}\left(x_{i}\right)=1+\sum_{l=x_{i}+1}^{L-1}w_{l}.$$

**Example 3.** 
*To illustrate the execution of the SQSMAS protocol, a concrete example is presented. Consider a trusted third party TP and n=3 participants $\left\{U_{1},U_{2},U_{3}\right\}$. The private input range is [0,4], i.e., L=5. The private inputs of the three participants are $x_{1}=1$, $x_{2}=3$, and $x_{3}=2$.*

*Each participant constructs an encoded vector $v_{i}=\left[v_{i0},v_{i1},\ldots ,v_{i4}\right]$ of length L, where $v_{ij}=1$ if $j=x_{i}$ and $v_{ij}=0$ otherwise. The corresponding vectors are $v_{1}=\left(0,1,0,0,0\right)$, $v_{2}=\left(0,0,0,1,0\right)$, and $v_{3}=\left(0,0,1,0,0\right)$.*

*All participants generate a shared random mask matrix $R=(r_{il})$ via the SQSMS protocol, satisfying*

(47) $$\sum_{i=1}^{3}r_{il}\equiv 0\;\left(\mathrm{mod}\;d\right).$$

*Set the modulus d=10, and the mask matrix is*

$$R=\left(\begin{array}{ccccc} 2 & 3 & 4 & 1 & 5\\ 5 & 4 & 1 & 6 & 2\\ 3 & 3 & 5 & 3 & 3 \end{array}\right).$$

Each participant encrypts its vector by $v_{il}^{*}=\left(v_{il}+r_{il}\right)\;\mathrm{mod}\;10$, obtaining encrypted vectors $v_{1}^{*}=\left(2,4,4,1,5\right)$, $v_{2}^{*}=\left(5,4,1,7,2\right)$, and $v_{3}^{*}=\left(3,3,6,3,3\right)$, which are sent to the TP.

*After receiving all the encrypted vectors the TP performs bitwise aggregation:*

$$w_{l}=\sum_{i=1}^{3}\left(v_{il}+r_{il}\right)\;\mathrm{mod}\;10.$$

*Since the column sum of masks is zero modulo d, the random masks cancel out automatically, and the true statistical result is W=(0,1,1,1,0), which indicates that each value 1, 2, and 3 appears once.*

*Subsequently, the TP publishes W to all participants, and each one calculates its ranking locally based on its private input. According to the ascending ranking formula ${\mathrm{Rank}}_{\mathrm{asc}}=1+\sum_{l=0}^{x_{i}-1}w_{l}$: Participant $U_{1}$ with $x_{1}=1$ obtains an ascending rank $1+w_{0}=1$; Participant $U_{3}$ with $x_{3}=2$ obtains an ascending rank $1+w_{0}+w_{1}=2$; Participant $U_{2}$ with $x_{2}=3$ obtains an ascending rank $1+w_{0}+w_{1}+w_{2}=3$.*

*According to the descending ranking formula ${\mathrm{Rank}}_{\mathrm{desc}}=1+\sum_{l=x_{i}+1}^{L-1}w_{l}$: $U_{2}$ gets the first rank, $U_{3}$ gets the second rank, and $U_{1}$ gets the third rank in descending order.*

*In the whole process, the TP only obtains encrypted data and publishes the aggregated statistical results. No participant can acquire the exact input value or identity information of others. The protocol preserves data privacy and identity anonymity while guaranteeing the correctness of the sorting results.*

**Correctness.** The correctness of the SQSMAS protocol stems from the unbiased aggregation property of the SQSMS protocol. Each participant encodes its private input into a unit vector, encrypts the vector with shared masks, and sends it to the TP. Since the masks satisfy $\sum_{i=1}^{n}r_{ij}\equiv 0\;\left(\mathrm{mod}\;d\right)$, the aggregation result calculated by the TP is $w_{l}=\sum_{i=1}^{n}v_{il}$, which accurately counts the occurrence frequency of each value. Based on the statistical vector, each participant can locally compute its exact ascending or descending ranking without recovering the original raw data.

**Security.** The SQSMAS protocol inherits the privacy-preserving characteristics of the SQSMS protocol. All uploaded data are encrypted by random masks and statistically independent of the original inputs. Thus, neither the TP nor external adversaries can recover the exact ownership of individual private inputs. Meanwhile, the TP only publishes the aggregated statistical vector rather than individual raw data. Each participant can only compute its own ranking with its private input and cannot deduce the specific values or identities of the other participants. Under the semi-honest and non-full-collusion model the uncertainty introduced by random masks guarantees the indistinguishability of private inputs and achieves a good balance between ranking availability and input privacy.

## 6. Conclusions

In this paper, we have proposed an efficient Semi-Quantum Secure Multi-party Summation protocol based on *d*-dimensional *n*-particle entangled states. Designed under the semi-quantum framework, the protocol generates cancelable random masks by exploiting the global correlation of entangled states to realize secure aggregation of private inputs. The entire computation relies only on quantum measurement operations, without requiring pre-shared keys or a multi-TP collaborative architecture. It improves communication efficiency and structural complexity while maintaining satisfactory security. Furthermore, we have conducted a formal analysis of the correctness and security of the protocol, and we constructed a corresponding quantum circuit model on the IBM Qiskit platform for simulation verification. Moreover, we extended the basic protocol to design application schemes for anonymous voting, auction and sorting, which demonstrate the good scalability and application potential of the proposed method.

Although the protocol achieves promising results in the research of Semi-Quantum Secure Multi-party Summation there still exists room for further improvement. First, the protocol exhibits relatively high dependence on the quantum capability of the trusted third party. In future work, adaptive mechanisms will be investigated to enable dynamic adaptation according to the available quantum resources of the TP, so as to reduce such dependence and enhance the flexibility and scalability of the protocol. In addition, the current scheme lacks global process simulation, making it difficult to comprehensively evaluate its practical performance. Future research could establish a global simulation model to analyze the protocol behavior under diverse scenarios, identify inefficient parts, and optimize the overall structure. In summary, subsequent research will focus on improving quantum resource utilization and conducting comprehensive scenario-based simulations so as to further refine the protocol and validate its computational performance and engineering feasibility.

## Figures and Tables

**Table 1:** List of Symbols.

Symbol	Definition
*n*	Number of participants
*d*	Modulus for modular arithmetic or dimension of the Hilbert space
δ	Number of decoy particles used for eavesdropping detection
$x_{i}$	Private input of participant $P_{i}$
|Φ〉	Original *d*-dimensional *n*-particle entangled state
|ψ〉	Entangled state after QFT
$r_{ij},r_{il}$	Shared random mask or measurement result
$c_{i}$	Ciphertext
*F*	Quantum Fourier transform operator
$S_{i}$	A sequence sent from TP to $P_{i}$
$S_{i}^{\prime }$	Sequence returned by $P_{i}$ after operator REFLECT or MEASURE
$f_{j}$	Bitwise aggregated value computed by TP
*R*	The summation result
η	Quantum communication efficiency
$W,w_{l}$	Aggregated statistical vector and its elements

**Table 2:** Eavesdropping detection criteria.

Case	QFT	Measurement Basis	Result
1	Yes	Computational basis	The sum of all measurement results modulo *d* equals 0
2	Yes	Fourier basis	All measurement results are identical
3	No	Computational basis	All measurement results are identical
4	No	Fourier basis	The sum of all measurement results modulo *d* equals 0

**Table 3:** Performance evaluation of SQSMS protocols.

Protocol	Quantum Resources	Summation Type	Quantum Operations	Pre-Shared Key	Number of TPs	Quantum Efficiency
Lian [34]	*d*-dimensional single-particle states	Modulo *d*	Measurement	No	2	$\frac{1}{25n+1}$
Ye [33]	*d*-dimensional *n*-particle entangled states, single-particle states	Modulo *d*	Measurement	No	1	$\frac{1}{2^{n}+2+\delta }$
Yi [25]	*d*-dimensional *n*-particle entangled states	Modulo *d*	SUM gate, $U_{k}$ gate, and measurement	No	1	$\frac{1}{n(L+1)}$
Tian [30]	*n*-qubit product states	Modulo 2	Measurement	Yes	1	$\frac{L}{2L+\delta }$
Our protocol	*d*-dimensional *n*-particle entangled states	Modulo *d*	Measurement	No	1	$\frac{d}{2d+\delta }$

*d*: Hilbert space dimension; *L*: length of private input encoding sequence; δ: number of decoy particles for eavesdropping detection; *n*: number of participants.

## Data Availability

No new data were created or analyzed in this study. Data sharing is not applicable to this article.
